# Hydroxyl-radical-induced oxidation of 5-methylcytosine in isolated and cellular DNA

**DOI:** 10.1093/nar/gku334

**Published:** 2014-05-22

**Authors:** Guru S. Madugundu, Jean Cadet, J. Richard Wagner

**Affiliations:** 1 Département de Médecine Nucléaire et Radiobiologie, Faculté de Médecine et des Sciences de la Santé, Université de Sherbrooke, 3001 12e Avenue Nord, Québec J1H 5N4, Canada; 2 Institut Nanosciences & Cryogénie/DSM, CEA/Grenoble, 38054 Grenoble, France

## Abstract

The methylation and oxidative demethylation of cytosine in CpG dinucleotides plays a critical role in the regulation of genes during cell differentiation, embryogenesis and carcinogenesis. Despite its low abundance, 5-methylcytosine (5mC) is a hotspot for mutations in mammalian cells. Here, we measured five oxidation products of 5mC together with the analogous products of cytosine and thymine in DNA exposed to ionizing radiation in oxygenated aqueous solution. The products can be divided into those that arise from hydroxyl radical (•OH) addition at the 5,6-double bond of 5mC (glycol, hydantoin and imidazolidine products) and those that arise from H-atom abstraction from the methyl group of 5mC including 5-hydroxymethylcytosine (5hmC) and 5-formylcytosine (5fC). Based on the analysis of these products, we show that the total damage at 5mC is about 2-fold greater than that at C in identical sequences. The formation of hydantoin products of 5mC is favored, compared to analogous reactions of thymine and cytosine, which favor the formation of glycol products. The distribution of oxidation products is sequence dependent in specific ODN duplexes. In the case of 5mC, the formation of 5hmC and 5fC represents about half of the total of •OH-induced oxidation products of 5mC. Several products of thymine, cytosine, 5mC, as well as 8-oxo-7,8-dihydroguanine (8oxoG), were also estimated in irradiated cells.

## INTRODUCTION

The methylation of cytosine in genomic DNA, which takes place mainly at CpG dinucleotides and constitutes 4–5% of the total cytosine, is an important epigenetic mark of gene expression. CpG are frequent hot spots in the mutation spectra in mice, rats and humans such that a majority of GC→AT transitions occur at CpG dinucleotides (1). About one-third of mutations in human genetic diseases appear to be associated with base substitutions at CpG dinucleotides (2). Between 25% and 50% of mutations in the tumor suppressor p53 gene of cancer cells are GC→AT transitions at CpG dinucleotides (2–4). Two types of epigenetic changes usually take place in cancer cells: (i) hypermethylation of CpG island promoter regions associated with transcriptional silencing of tumor-suppressor genes (e.g. *MLH1*, *BRCA1*, *MGMT*), and (ii) global hypomethylation, i.e. loss of methylation (20–60% genome-wide) particularly within repetitive genomic sequences (5). The reason for such hypermutability of CpG dinucleotides remains unknown. One hypothesis suggests that 5-methylcytosine (5mC) is more susceptible to thermal deamination than cytosine. The difference in the rate of thermal deamination of cytosine and 5mC is about 4-fold for the monomer or single-stranded DNA (half-life = 50 and 200 y, respectively) and between 2- and 3-fold for double-stranded DNA (half-life = 38 000 and 85 000 y, respectively) (6–10). However, the difference in the thermal deamination of cytosine and 5mC in double-stranded DNA (2–3-fold) does not explain the difference in mutability of methylated and nonmethylated CpG (12–42-fold) (2). Another hypothesis is that the repair of G:U base pairs by uracil DNA glycosylase (UNG) is more efficient than the repair of G:T base pairs (11). As a complement to these hypotheses, we propose that CpG hypermutability is due to the pathway of oxidation and the subsequent chemistry of final products.

Recent interest in 5mC arises from the discovery of extraordinarily high levels of 5-hydroxymethylcytosine (5hmC), an oxidation product of 5mC, in the DNA of brain Purkinje and granule cells (12). The levels of 5hmC range from 0.1 to 0.7% of cytosine depending on the tissue (13,14). The oxidation of 5mC to 5hmC in cellular DNA involves the ten-eleven translocation (TET1, 2 and 3) family of enzymes, which are iron (II)- and α-ketoglutarate-dependent dioxygenases present in the nucleus. Iterative oxidation also occurs to produce 5-formylcytosine (5fC) and 5-carboxylcytosine (15–17). Thereby, these enzymes have been shown to oxidize 5mC *in vitro*, reduce the level of 5hmC in TET null cells, and moreover, the expression of TET enzymes correlates with the differentiation of embryonic cells during development (18,19). Thus, TET enzymes initiate the first step in the demethylation of methylated 5mCpG dinucleotides. Other steps of demethylation may follow including deamination of 5hmC by AID/APOBEC, removal of 5hmU by thymine DNA glycosylase (TDG) and single-strand specific monofunctional DNA glycosylase 1 (SMUG1) to complete the demethylation of 5mC to cytosine (20,21). Interestingly, the levels of 5hmC in the DNA of cancer cells, e.g. glioblastoma, myeloid malignancies, etc. are considerably lower than those observed in normal cells (22,23).

**Figure 1.**
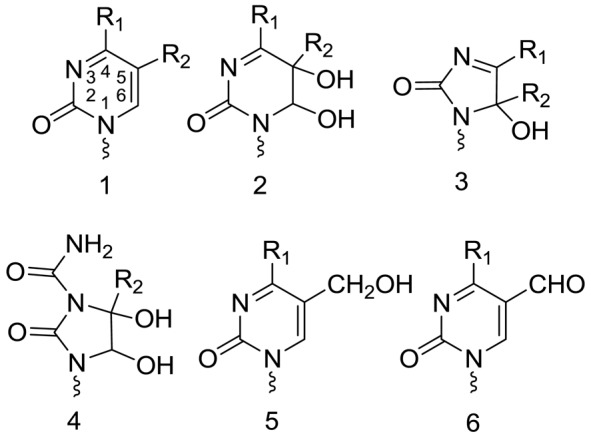
Structures of pyrimidine oxidation products. Each structure includes derivatives with different substituents for R_1_ and R_2_: 5-methylcytosine (5mC (**1a**): R_1_ = NH_2_;R_2_ = CH_3_), thymine (T (**1b**): R_1_ = OH;R_2_ = CH_3_), cytosine (C (**1c**): R_1_ = NH_2_;R_2_ = H) and uracil (U (**1d**): R_1_ = OH;R_2_ = H). Abbreviations for the different products are denoted by the nucleobase-modification, e.g. 5mC-Gly (**2a**) is the glycol product of 5-methylcytosine, T-Hyd (**3b**) is the hydantoin product of thymine, C-Imid (**4c**) is the imidazolidine product of C, etc. The 5-hydroxymethyl derivatives are denoted as 5hmC (**5a**) and 5hmU (**5b**) and the 5-formyl derivatives as 5fC (**6a**) and 5fU (**6b**). Pyrimidine bases are attached by a squiggly line indicating that they are either attached to a 2-deoxyribose moiety (i.e. 2′-deoxyribonucleosides) or are part of a DNA chain (Figure 2).

Here, we apply liquid chromatography tandem mass spectrometry (LC-MS/MS) to measure five oxidation products of 5mC (**1a**) as modified 2′-deoxyribonucleosides: 5,6-dihydroxy-5,6-dihydro-5-methyl-2′-deoxycytidine (5mC-Gly, **2a**); 5-hydroxy-5-methyl-4-aminohydantoin (5mC-Hyd, **3a**); 1-carbamoyl-3,4-dihydroxy-2-oxo-imidazolidine (5mC-Imid, **4a**); 5-hydroxymethylcytosine (5hmC, **5a**) and 5-formylcytosine (5fC, **6a**; see structures in Figure 1). In addition, the analogous products arising from the oxidation of cytosine (five products) and thymine (four products) in DNA are monitored in the same analysis. The formation of these products is examined in five oligonucleotide (ODN) duplexes containing different amounts of 5mC, calf-thymus (CT) DNA, and intact mammalian cells exposed to ionizing radiation. These analyses demonstrate that 5mC is more sensitive than nonmethylated C to •OH-induced oxidation. The major products of 5mC oxidation include 5mC-Hyd (**3a**) and 5fC (**6a**) while the formation of 5mC-Gly (**2a**), 5mC-Imid (**4a**) and 5hmC (**5a**) is minor.

## MATERIALS AND METHODS

All chemicals were purchased from Sigma-Aldrich and were of reagent grade unless stated otherwise. The purification of modified nucleosides was carried out by HPLC-UV using an Alliance system (Waters 2795 or 2690) connected to dual wavelength UV detectors (Waters 2487) and controlled by Millenium workstation (Waters version 4). For HPLC analysis with UV detection, the separation of modified nucleosides was achieved on a reversed phase column (5 μm ODS A 250 × 6 mm; YMC) using isocratic conditions or a gradient from 100% of mobile phase A (ammonium formate (20 mM) adjusted to pH 5) and 0–20% of mobile phase B (90% acetonitrile) with a flow rate of 1 ml/min. Melting temperatures (*T*_m_) of ODN duplexes were determined on a spectropolarimeter (Jasco J-810) at 250 nm and scan rate of 1°C per min (10–95°C). The experimental melting curves were fit to a two-state transition using graphical van't Hof analysis (24,25). ^1^H-NMR analyses were performed in D_2_O using Bruker 300 MHz and Varian 600 MHz spectrometers.

### Irradiation of oligonucleotides, calf-thymus DNA and F98 cultured cells

**Figure 2.**
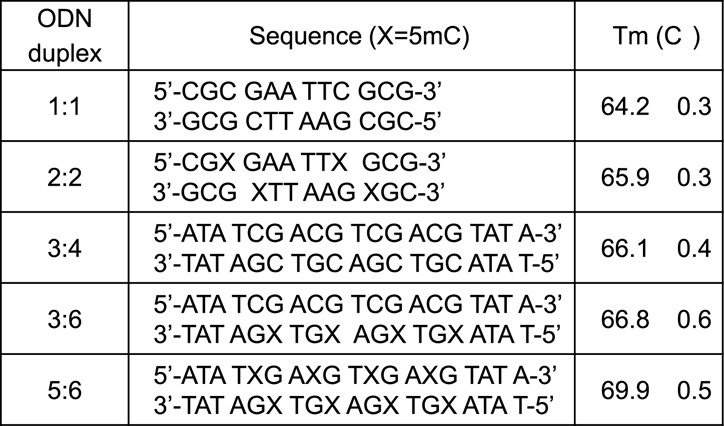
Sequences and melting temperatures of ODN duplexes.

Oligonucleotides (ODNs; Figure 2) were purified before oxidation by reversed phase HPLC using a gradient of formate buffer (20 mM) to the same buffer containing 30% acetonitrile in 25 min at a flow rate of 1 ml/min using a YMC-Pack Pro C18 column (250 length × 6.0 mm I.D.; particle size = 5 µm). The ODNs eluted between 12 and 15 min under these conditions. The purified ODNs were subsequently desalted by passage through a 3 kDa molecular weight filtering device (Amicon Ultra, Ultracel-3K), which lead to a dilution of salt >100-fold (<0.2 mM). The purified ODNs were dissolved in 50 mM NaCl and 20 mM phosphate buffer (pH 6.9) and they were annealed with their complementary strand by heating to 90°C for 5 min and allowing them to cool to 23°C over a period of 2 h. ODN duplexes and CT-DNA solutions were bubbled 10 min prior to irradiation as well as during irradiation with a continuous and pre-hydrated flow of oxygen gas. ODN duplexes and CT-DNA (1.2 mg/ml) were irradiated in an open 1.5 ml Eppendorf tube placed in the center of a Gammacell 220 delivering 1.2 Gy/min for 0–2 h (0–200 Gy). The radiation dose was determined by Fricke dosimetry as previously described (26). After irradiation, the duplex ODNs were precipitated by the addition of 3 M sodium acetate (final concentration 0.3 M) and 2.2 volumes of ice cold ethanol followed by centrifugation at 14 000 g at 4ºC for 30 min. The DNA pellet was rinsed twice with 90% ethanol.

Experiments with cells were carried out using cultured Fischer glioma cells (F98) obtained from ATCC (Manassas, VA). Cells were grown in monolayers using Dulbecco's modified Eagle's medium supplemented with 10% fetal bovine serum (Gibco, Burlington, ON), 26.2 mM of sodium bicarbonate, 2 mM L-glutamine and a mix of penicillin (100 μl/ml) and streptomycin (100 μl/ml) at 37°C in a humidified environment with 5% CO_2_. The cells were washed with phosphate buffered saline prior to irradiation. The cells were pelleted by centrifugation at 1000 g and excess liquid was carefully removed from the top of the cells. F98 cells were then irradiated in a closed 1.5 ml Eppendorf tube placed in the center of a Gammacell (ELAN 3000, Theratronics, Ottawa, ON) emitting γ-ray from a Cs-137 source (1.2 MeV) at a dose rate of 11.30 Gy/min (irradiation time = 0–6 h) as determined by Fricke dosimetry (26).

### Extraction of DNA from irradiated F98 cells

After irradiation, F98 cells (∼5 million) were suspended in 300 μl of extraction buffer (10 mM Tris-Cl (pH 8.0) + 5 mM EDTA-Na_2_, 0.15 mM desferrioxamine pH 8.0) and subjected to two freeze-thraw cycles in liquid N_2_. Total RNA was removed by digestion at 50°C for 30 min with 20 μl of RNAse A (prepared in 1 mg/ml in 10 mM Tris-Cl (pH 8), 1 mM EDTA-Na_2_, 2.5 mM desferrioxamine pH 7.4). Then, 15 μl of protease (20 mg/ml in water) was added to the samples and incubated at 37°C for 120 min with frequent vortex. After protease treatment, DNA was precipitated by the addition of 600 μl of NaI solution (7.6 M NaI, 40 mM Tris-Cl (pH 8.0), 20 mM EDTA-Na_2_ and 0.3 mM desferrioxamine) and 1 ml of 2-propanol. The sample was kept at −20°C for 30–60 min and followed centrifugation at 14 000 g for 30 min at 4°C. The supernatant was removed and the pellet washed with 1 ml of 40% 2-propanol followed by 70% ethanol. The pellet was briefly dried under vacuum and stored in the freezer.

### Enzymatic digestion of DNA

DNA samples were dissolved in 40 μl of water and 5 μl of labeled internal standard mixture was added. For DNA digestion, 5 μl of 500 mM sodium acetate at pH 5.5 and 5 μl (5 units) of P1 nuclease (Sigma N8630, stock solution is prepared by the addition of 1 mg of lyophilized powder dissolved in 300 mM sodium acetate at pH 5.3) were added, and the solution was incubated at 37°C for 60 min. The resulting mixture of nucleotides was digested further by the addition of 10 μl Tris-Cl (500 mM, pH 8.0), 5 μl (0.005 units) of snake venom phosphodiesterase (Sigma P3134, stock solution is prepared by dissolving 9 mg of lyophilized powder in 100 μl of 50 mM Tris-Cl at pH 8.0) and 5 μl (5 units) of alkaline phosphatase (Roche, stock solution used as received in accompanied buffer solution). The mixture was vortexed and incubated at 37°C for 60 min. At the end of enzymatic digestion, 10 μl of phosphoric acid (0.22 M) and 40 μl of chloroform were added to the sample to remove excess protein by centrifugation at 10 000 rpm for 10 min. The supernatant (∼80 μl) was carefully withdrawn for LC-MS/MS analyses. The efficiency of enzymatic digestion was considered to be quantitative because adding additional enzyme or extending the time of incubation did not increase the release of damage as 2′-deoxyribonucleosides. Moreover, there was no evidence for the presence of nondigested dinucleotides containing damage in the final mixture as inferred by LC-MS/MS analysis (27,28).

### Synthesis of isotopically labeled standards

**Scheme 1.**
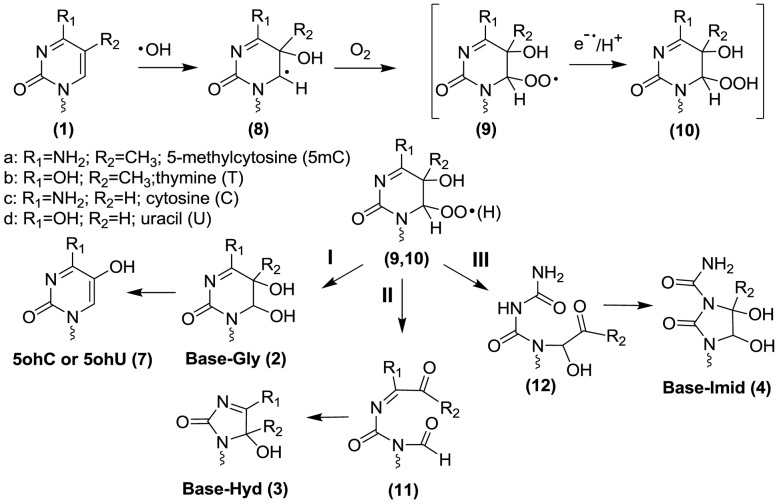
Proposed mechanism of formation of glycol, hydantoin and imidazolidine products.

Labeled thymidine (^15^N_2_; 96–98%), 2′-deoxycytidine (^15^N_3_; 96–98%) and 8-oxo-7,8-dihydro-2′-deoxyguanosine (^15^N_5_; 96–98%) were purchased from Cambridge Isotope Laboratories Inc. (Tewsbury, MD). The synthesis of natural and labeled standards for the oxidation products of 2′-deoxycytidine (U-Gly (**2d**), U-Hyd (**3d**), C-Imid (**4c**), 5ohC (**7c**), 5ohU (**7d**), and thymidine (T-Gly (**2b**), T-Hyd (**3b**), 5hmU (**5b**) and 5fU (**6b**) was carried out as described previously (28); structures **1–6** are shown in Figure 1 whereas structure **7c** and **7d** are shown in Scheme 1. 5-Methyl-2′-deoxycytidine was synthesized in three steps from labeled thymidine (^15^N_2_; 96–98%) and ammonium hydroxide (NH_4_–^15^N; 99%) using established procedures (29–31). The synthesis of 5mC-Hyd (**3a**) was carried out by ozonolysis using the same procedure as that for the synthesis of T-Hyd (**3b**) and U-Hyd (**3d**) (28). Likewise, the synthesis of 5mC-Imid was carried out by the same procedure as that for C-Imid via peroxidation of the 5-bromo-6-hydroxy-5,6-dihydro-5-methyl-2′-deoxycytidine (28,32). All labeled compounds were purified by reversed phase HPLC, and their identity was confirmed by MS analyses (28). MS analysis of 5mC oxidation products (**2a**–**6a**, 2′-deoxyribonucleosides) is provided in Supplementary Figures S2–S6.

**Figure 3.**
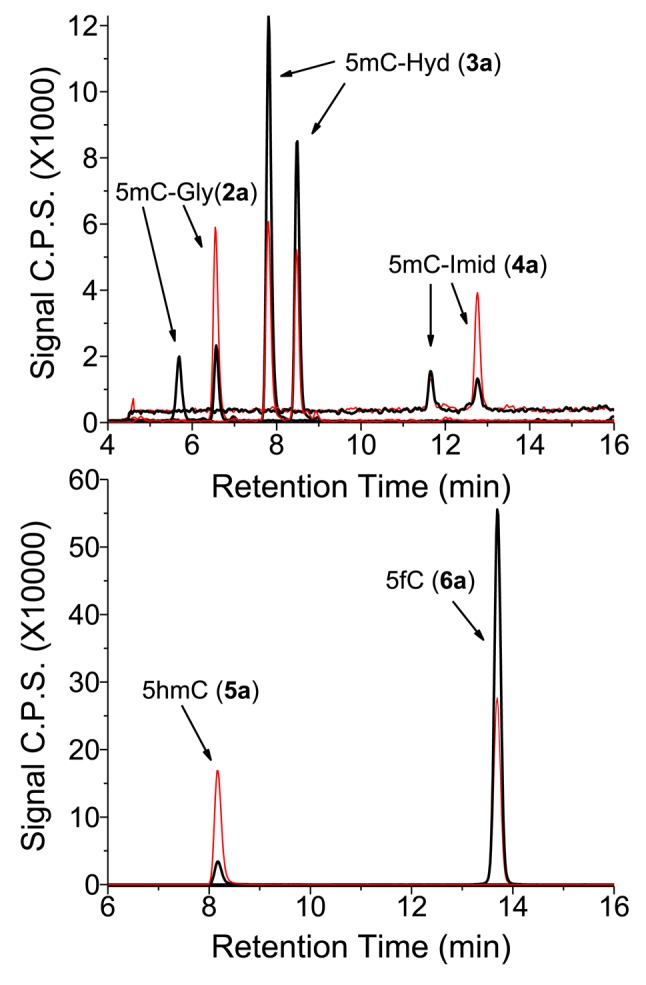
LC-MS/MS analysis of 5-methylcytosine (5mC) oxidation products in DNA. ODN 5:6 duplex was exposed to 50 Gy of ionizing radiation in oxygenated aqueous solution. The sample was digested enzymatically to its component 2′-deoxyribonucleosides. The equivalent of 35 μg of digested DNA was injected onto a Hypercarb column (top panel) for the analysis of 5mC-Gly (**2a**), 5mC-Hyd (**3a**) and 5mC-Imid (**4a**), and as well onto a reversed phase column (bottom panel) for the analysis of 5hmC (**6a**) and 5fC (**5a**). The natural products are shown in black and the isotopic standards in red. The amount of isotopic standards injected for **2a**, **3a** and **4a** was 3.0 pmol while that for **5a** and **6a** was 8.3 pmol.

**Figure 4.**
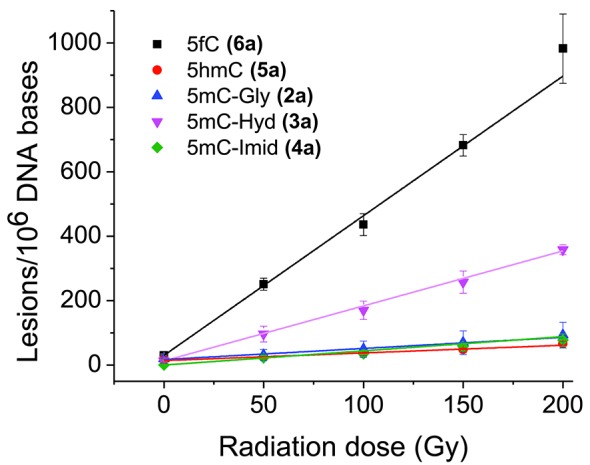
Rate of formation of oxidation products from isolated DNA. The formation of products of ODN 5:6 duplex was monitored as a function of radiation dose (Figure 3). The rate of formation was calculated from a linear fit of the points (*r*^2^ > 0.95). Results obtained from three independent experiments (*n* = 3).

**Figure 5.**
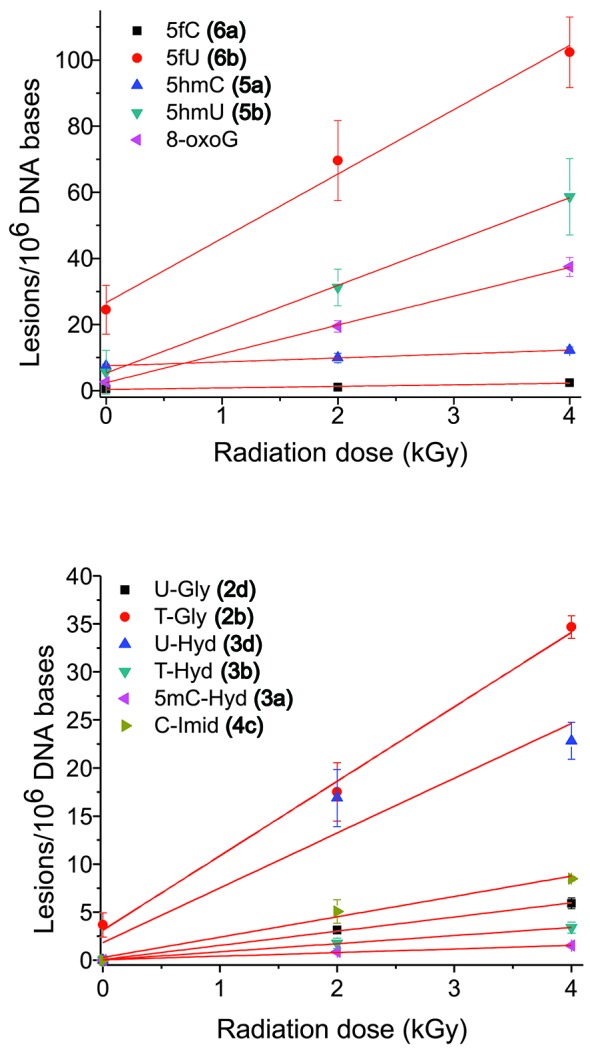
Rate of formation of oxidation products from cellular DNA. F98 cells were exposed to ionizing radiation (0–4 kGy), DNA was extracted and digested, and the modified 2′-deoxyribonucleosides were quantified by LC-MS/MS analyses. The formation of 5,6-unsaturated products is shown in the upper panel and that of saturated products in the lower panel. Results obtained from five independent experiments (*n* = 5). The values for the rate of formation of each product in cellular DNA are given in Table 2.

### LC-MS/MS analysis

The analysis of modified nucleosides was carried out using a conventional HPLC-UV system (Shimadzu LC-10AD pumps, DGU-10A degasser, SIL-HTc autoinjector, CTO-10AS column heater, SPD-20A UV detector) coupled to a tandem-mass spectrometer (MS/MS; API 3000 with Turbo Ionspray; AB-Sciex, Concord, Canada). The analysis of products with a saturated or fragmented pyrimidine ring, which includes glycol products (**2a**,**2b**,**2d**), hydantoin products (**3a**,**3b**,**3d**) and imidazolidine products (**4a**,**4c**), was carried out using a so-called Hypercarb column (250 length × 2.1 mm I.D.; particle size 5 μm; Thermo Scientific), whereas the 5,6-unsaturated products (**5a**,**5b**,**6a**,**6b**), 5-hydroxycytosine (5ohC (**7c**) and 5-hydroxyuracil (5ohU (**7d**)) were separated on an octadecylsilyl silica gel (ODS) column (250 length × 2.0 mm I.D.; particle size = 5 μm; YMC). In the first case (Hypercarb packed column), the products were eluted using a gradient starting with 90% ammonium formate (5 mM, pH 5) in 10% acetonitrile going to 65% of the latter buffer in 35% acetonitrile in 15 min at a total flow rate of 0.2 ml/min. The duration of analysis was 35 min, which included the gradient program for separation (15 min), column wash with 90% acetonitrile (10 min) and equilibration for the next injection (10 min). In the second case (ODS packed column), the products were eluted using a gradient starting with 95% ammonium formate (5 mM, pH 5) in 5% acetonitrile going to 80% of the latter buffer in 20% acetonitrile in 15 min at a total flow rate of 0.18 ml/min. The same wash program was used as above with the ODS column. The flow was split to the MS/MS instrument (∼85%) and the UV detector (∼15%) by a low volume union-tee. The products were detected by positive ionization using multiple reaction monitoring (MRM). The yield of products was determined from the ratio of specific ion signals for the natural and synthetic isotopic standards (added before DNA digestion). A mixture of isotopic standards and nonmodified 2′-deoxycytidine was injected every 3–4 samples in order to determine the amount of 2′-deoxycytidine in digested DNA by UV detection at 260 nm. The MRM signal of modified products in pure solution was linear over the observed levels of products in DNA samples (Supplementary Figures S2.2–S6.2). The calibration curves for the quantification of 2′-deoxycytidine and thymidine oxidation products was as previously reported (28).

## RESULTS

### General considerations

The melting curves of each ODN duplex were determined under the same conditions of salt and DNA concentration as the irradiations (Figure 2). The percentage of single-stranded DNA can be estimated by further analyses, which indicate that, during irradiation at 23°C, the contribution of single-stranded DNA was minor for the 12-mer ODN duplexes (estimated to be 3.3% for 1:1 and 6.7% for 2:2; Supplementary Figures S1.1 and S1.2) and lower for the 19-mer ODN duplexes (estimated to be between 0.1 and 1.5%; Supplementary Figures S1.3–S1.5). Thus, ODN molecules were predominantly double stranded during irradiation. Three model systems were chosen to examine DNA damage: the Dickerson-Drew dodecamer, a symmetrical 19-mer with four repeating CG sequences, and CT-DNA (Figure 2). The complete analysis of pyrimidine oxidation products was achieved by separate reversed phase and porous graphitic carbon ‘Hypercarb’ liquid chromatography coupled to tandem mass spectrometry (Figure 3). Fifteen modifications of DNA were measured as a function of radiation dose (0–200 Gy) and the corresponding rate of formation determined by linear regression of yield-dose graphs (Figure 4).

**Table 1.** Radiation-induced base damage in naked DNA

Oxidation products	ODN1:1^a^ (C)		ODN2:2^a^ (C+5mC)		ODN3:4^a^ (C)		ODN3:6^a^ (C+5mC)		ODN5:6^a^ (5mC)		CT-DNA^a^	
		%		%		%		%		%		%
5mC-Gly (**2a**)	—–^b^		1.49 ± 0.10	12	—–^b^		0.23 ± 0.04	8	0.43 ± 0.04	6	0.27 ± 0.02	26
5mC-Hyd (**3a**)	—–^b^		3.28 ± 0.23	26	—–^b^		0.77 ± 0.05	27	1.70 ± 0.12	23	0.27 ± 0.03	26
5mC-Imid (**4a**)	—–^b^		1.88 ± 0.13	15	—–^b^		0.17 ± 0.02	6	0.38 ± 0.10	5	<0.02^c^	0
5hmC (**5a**)	—–^b^		0.55 ± 0.04	4	—–^b^		0.08 ± 0.01	3	0.24 ± 0.05	3	0.03 ± 0.01	3
5fC (**6a**)	—–^b^		5.45 ± 0.38	43	—–^b^		1.56 ± 0.44	56	4.67 ± 0.47	63	0.45 ± 0.03	44
Total 5mC (**1a**)			12.65 ± 0.89	100			2.81 ± 0.56	100	7.42 ± 0.78	100	1.04 ± 0.09	100

U-Gly (**2d**)	1.35 ± 0.09	11	1.44 ± 0.10	17	0.76 ± 0.10	19	0.52 ± 0.10	22	—–^b^		0.83 ± 0.06	14
U-Hyd (**3d**)	1.88 ± 0.13	15	1.17 ± 0.08	14	1.32 ± 0.39	34	0.72 ± 0.09	30	—–^b^		1.08 ± 0.08	18
C-Imid (**4c**)	5.38 ± 0.38	43	2.15 ± 0.15	25	0.48 ± 0.04	12	0.28 ± 0.07	11	—–^b^		1.23 ± 0.15	21
5ohC (**7c**)	2.38 ± 0.17	19	2.28 ± 0.16	27	0.54 ± 0.09	14	0.31 ± 0.07	13	—–^b^		1.47 ± 0.10	25
5ohU (**7d**)	1.48 ± 0.10	12	1.53 ± 0.11	18	0.80 ± 0.07	21	0.57 ± 0.05	24	—–^b^		1.27 ± 0.09	22
Total C (**1c**)	12.47± 0.87	100	8.57 ± 0.60	100	3.90 ± 0.69	100	2.40 ± 0.38	100			5.88 ± 0.48	100

T-Gly (**2b**)	1.69 ± 0.12	17	1.43 ± 0.10	18	2.60 ± 0.20	32	2.68 ± 0.38	32	3.31 ± 0.23	29	1.89 ± 0.13	16
T-Hyd (**3b**)	0.45 ± 0.03	5	0.16 ± 0.01	2	0.56 ± 0.07	7	0.45 ± 0.11	5	0.60 ± 0.04	5	0.53 ± 0.04	4
5hmU (**5b**)	0.51 ± 0.04	5	0.87 ± 0.06	11	0.42 ± 0.05	5	0.33 ± 0.02	4	0.55 ± 0.14	5	0.95 ± 0.07	8
5fU (**6b**)	7.21 ± 0.50	73	5.38 ± 0.38	69	4.65 ± 0.60	56	4.95 ± 0.35	59	6.79 ± 0.48	60	8.70 ± 0.61	72
Total T (**1b**)	9.86 ± 0.69	100	7.84 ± 0.55	100	8.23 ± 0.92	100	8.41 ± 0.86	100	11.25 ± 0.89	100	12.07± 0.85	100

8oxoG	40.8 ± 2.9		18.6 ± 1.3		14.4 ± 1.2		11.8 ± 2.8		16.2 ± 1.1		11.8 ± 0.8	

^a^Yields are given in units of lesions/million DNA bases. They were obtained from the rate of formation of lesions as a function of radiation exposure (Figure 4). The initial concentration of ODN duplexes and CT-DNA was 0.80 mg/ml. The S.D. for total damage was taken as the sum of individual S.D. for each product.

^b^Not detected because the parent DNA base was absent.

^c^A small increase above baseline was observed at the highest dose.

5mC products include the corresponding glycol (**2a**), hydantoin (**3a**) and imidazolidine (**4a**) products together with 5hmC (**5a**) and 5fC (**6a**). A complication in the analysis of 5mC-Gly products was their deamination to T-Gly during enzymatic digestion of DNA. For the quantification of 5mC-Gly, we used one of the two isotopic standards that are separated using ‘Hypercarb’ chromatography. The assumption that both isomers of 5mC-Gly give the same response by MS is reasonable in view of the equal MRM signal observed in chromatograms (Figure 3) and the reported close similarities between isomers of pyrimidine glycols (33). The two isomers of 5mC-Gly also give approximately equal signals using alternative MRM transitions (*m/z* 276 to 117 (loss of the base moiety), *m/z* 276 to 258 (loss of H_2_O), *m/z* 276 to 160 (loss of the sugar moiety) and *m/z* 276 to 142 (loss of the sugar and H_2_O)). The deamination of 5mC-Gly to T-Gly has been reported to take place with half-lives of 5mC-Gly of about 20 h (17 and 22 h) in aqueous neutral solution at 37°C as 2′-deoxyribonucleosides and of about 32 h (27 and 37 h) under the same conditions in duplex DNA (34,35). Based on changes in the amount of isotopic standard of 5mC-Gly, we estimate that 20% of 5mC-Gly undergoes deamination during enzymatic digestion. In addition to 5mC-Gly, 5mC-Hyd (**3a**) undergoes deamination to T-Hyd (**3b**) during enzymatic digestion albeit to a lesser extent (<5%) than the above reaction for 5mC-Gly. Thus, the yields of T-Gly and T-Hyd were corrected accordingly. In contrast to 5mC-Gly (**2a**) and 5mC-Hyd (**3a**), the corresponding oxidation products of cytosine, C-Gly (**2c**) and C-Hyd (**3c**) are very unstable as 2′-deoxyribonucleosides in neutral aqueous solutions at 37°C (half-life < 1 h) (36,37). Thus, we assume that they convert quantitatively during DNA digestion to the corresponding uracil derivatives, U-Gly (**2d**) and U-Hyd (**3d**), respectively (in this case, the isotopic standards of the deaminated products were used for quantification). The lack of a CH_3_ substituent at C5 in the case of cytosine allows for the efficient dehydration of initial C-Gly (**2c**) products to 5ohC (**7c**) and 5ohU (**7d**). Although the glycols of 5mC and thymine are stable enough for quantification in DNA, the corresponding products of cytosine transform to a mixture of U-Gly (**2d**), 5ohC (**7c**) and 5ohU (**7d**).

### Effect of cytosine methylation in identical sequences

To determine the effect of methylation on DNA damage, two internal cytosine (C) residues were replaced with 5mC (ODN 1:1 and ODN 2:2). The effect of methylation on DNA damage was also determined in duplexes containing 19 base pairs with four repeating CG sequences (Figure 2): nonmethylated (ODN 3:4), hemimethylated (ODN 3:6) or fully methylated (ODN 5:6). Lastly, purified CT-DNA was used as a common model system for DNA damage assessment. Table 1 summarizes the observed rate of formation of oxidation pyrimidine products upon exposure of ODN duplexes and CT-DNA to gamma-radiation. The effect of methylation on DNA damage was ascertained from the total damage at C and 5mC sites in DNA (Table 1). For the short ODNs (ODN 1 and 2, self-complementary 12-mer), the total of C damage in ODN 1:1 with four C residues was 12.7, whereas the total of C and 5mC damage in ODN 2:2 with two residues each of C and 5mC increased to 21.2 in units of damage per million DNA bases. Similarly, the total damage at C and 5mC sites increased from 3.9 to 5.2 to 7.4 damage per million DNA bases when C was progressively replaced with 5mC in the series of ODN duplexes containing C only (ODN 3:4), equal amounts of C and 5mC (ODN 3:6), and only 5mC (ODN 5:6), respectively (Table 1). These results clearly demonstrate that when C is replaced with 5mC in DNA at the exact same position in DNA sequences, the total damage at that site increases by an average of 1.8-fold.

The above assessment can also be extended to CT-DNA. In this case, the effect of sequence is averaged throughout the genome of CT-DNA. Assuming that •OH reacts equally with C and 5mC, the difference in damage at C and 5mC should reflect the amount of each of these bases in DNA. Hence, one can calculate as a first estimation that damage at C will be 15.6-fold (100/6.4) higher than damage at 5mC based on the known percentage of 5mC to C in CT-DNA (6.4%; (38,39)). In comparison, the total damage at C was 5.9 lesions/10^6^ bases and that at 5mC was 1.0 lesions/10^6^ bases in CT-DNA (Table 1), giving a difference of 5.7-fold (5.9/1.0). The difference between expected and observed damage in CT-DNA is 2.7 (15.6/5.7), which is higher than that observed for ODNs but nevertheless in agreement with the estimated greater sensitivity of 5mC to •OH-induced damage observed for ODNs.

### Distribution of products for 5mC, cytosine and thymine in isolated DNA

The distribution of oxidation products of 5mC was 9%, 25% and 9% for 5mC-Gly (**2a**), 5mC-Hyd (**3a**) and 5mC-Imid (**4a**), and 54% and 3% for 5hmC (**5a**) and 5fC (**6a**), respectively (calculated from the absolute yields given in Table 1, taking the average of three duplexes (ODN 2:2; ODN 3:6; ODN 5:6)). The yields of 5mC products were similar in ODN duplexes and CT-DNA except for 5mC-Gly (**2a**) that increased significantly in CT-DNA (9% (average of duplexes) to 26%). In the case of cytosine, the percentage of damage in ODN duplexes was fairly evenly distributed over five major products: U-Gly (**2d**; 17%), U-Hyd (**3d**; 23%), C-Imid (**4c**; 23%), 5ohC (**7c**; 18%) and 5ohU (**7d**; 19%) (taking the average of four ODN duplexes). Likewise, the distribution of C oxidation products was similar in ODN duplexes and CT-DNA. Lastly, total damage to thymine in ODN duplexes can be divided into four major products: T-Gly (**2b**; 26%), T-Hyd (**3b**; 5%), 5hmU (**5b**; 6%) and 5fU (**6b**; 63%). In comparison, 5fU appeared to increase at the expense of T-Gly in CT-DNA. There were several interesting changes in the distribution of base products in ODN duplexes. For example, the percentage of 5fC significantly increased (43% to 63%) while 5mC-Imid decreased (15% to 5%) in ODN 5:6 compared to ODN 2:2; the percentage of 5ohC increased (19% to 27%) and C-Imid decreased (43% to 25%) in ODN 1:1 compared to ODN 2:2. Surprisingly, the yield of 8oxoG varied remarkably among the five ODN duplexes (12 to 41 lesions/million DNA bases).

### Formation of 5mC, C and T oxidation damage in cellular DNA

The formation of products was measured in cells exposed to ionizing radiation (2 and 4 kGy). It was necessary to apply lethal doses of radiation to reach the appropriate levels of damage for their quantification by LC-MS/MS (Figure 5, Table 2). The radiation-induced rate of formation for T-Gly, 5fU, 5hmU and 8oxoG were comparable to the values reported previously using LC-MS/MS (40–43). In some cases, there was a relatively high amount of damage in the DNA of non-irradiated cells. This damage can be attributed to either endogenous or artificial damage induced during the extraction of DNA from cells and subsequent procedures. For example, the high amount of 8oxoG (2.5 lesions/10^6^ bases) in non-irradiated cells likely arises by autooxidation of guanine during sample preparation in view of the generally lower level of this damage that has been previously observed in cellular DNA (0.5–1.2 lesions/10^6^ bases) (44,45). In contrast, the relatively high amount of 5hmC (7.5 lesions/10^6^ bases) and 5fC (0.5 lesions/10^6^ bases) in non-irradiated cells is most likely due to the enzymatic oxidation of 5mC by TET family enzymes (13,14,46). Although 5-hydroxycytosine (5ohC) and 5-hydroxyuracil (5ohU) were detected in ODN duplexes and isolated DNA exposed to ionizing radiation, the level of these products was below our detection limit from cellular DNA. In the case of 5ohC and 5ohU, however, the recovery of isotopic standard was poor during DNA digestion, suggesting the presence of impurities that may selectively destroy these modified bases that have a low oxidation potential (47).

**Table 2.** Radiation-induced base damage in cellular DNA

Product/DNA base^a^	5mC (**1a**)	C (**1c**)	T (**1b**)	Guanine
Gly (**2**)	n.d.	1.48 ± 0.14	7.75 ± 0.10	
Hyd (**3**)	0.37 ± 0.01	5.71 ± 0.48	0.85 ± 0.14	
Imid (**4**)	n.d.^b^	2.12 ± 0.06		
5hmC/5hmU (**5**)	1.20 ± 0.14		13.26 ± 1.64	
5fC/5fU (**6**)	0.47 ± 0.05		19.47 ± 1.15	
8oxoG				8.72 ± 0.72

^a^Rate of formation of pyrimidine oxidation products in cellular DNA in units of lesions per 10^6^ non-modified DNA bases per kGy of ionizing radiation. The values were obtained from the graphs presented in Figure 5. Levels of modifications in non-irradiated cells were 7.5 5hmC, 0.5 5fC, 5.6 5hmU, 24.5 5fU, 3.7 T-Gly and 2.5 8oxoG lesions per 10^6^ nonmodified DNA bases modifications; the other lesions were not detected in non-irradiated cellular DNA (<0.1).

## DISCUSSION

### Mechanism of oxidation of pyrimidine bases

**Scheme 2.**
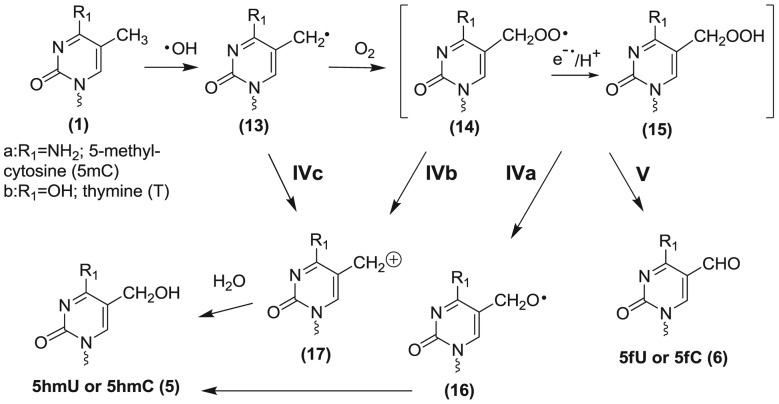
Proposed mechanism of formation of methyl products of thymine and 5-methylcytosine.

The proposed mechanism of oxidation of 5mC is depicted in Schemes 1 and 2. The mechanism has been adapted to include analogous products of cytosine and thymine. When an aqueous dilute solution of DNA is exposed to ionizing radiation, the main reactive species is •OH, which rapidly undergoes addition to the 5,6-double bond of pyrimidine bases and abstracts a hydrogen atom from the methyl group of thymine and 5mC. The reaction of solvated electrons (e_aq_) with DNA is negligible because these species predominantly react with oxygen to generate superoxide radical anions (O_2_^•−^) in oxygenated solutions. It may be added that pyrimidine base radical anions that arise from the addition of e_aq_ are easily oxidized to the parent nucleobases with the release of unreactive O_2_^•−^. The reaction of •OH with pyrimidine bases takes place at both C5 and C6 with a bias toward C5, leading to the formation of intermediate reducing pyrimidin-6-yl radicals (**8**, Scheme 1, intermediate oxidizing pyrimidin-5-yl radicals are not shown). In the presence of O_2_, pyrimidin-6-yl radicals (**8**) convert to the corresponding peroxyl radicals (**9**). The fate of pyrimidine peroxyl radicals in DNA has been proposed to involve addition reactions to pyrimidine or purine bases, H-atom abstraction with neighboring 2-deoxyribose moieties, and electron transfer to guanine although the latter reaction has been recently questioned (48–52). It is reasonable to assume that the subsequent decomposition reactions of peroxyl radicals in DNA involve the formation of either a hydroperoxide or the putative formation of an endoperoxide between two DNA bases. In the case of thymidine 5(6)-hydroxy-6(5)-hydroperoxides (**10**) (53), the decomposition pathway can be explained by O–O bond cleavage to give glycol products (**2**; Pathway I), C5-C6 bond cleavage to give an intermediate (**11**) that transforms into hydantoin products (**3**; Pathway II), and C4-C5 bond cleavage to give an intermediate (**12**) that transforms into imidazolidine products (**4**; Pathway III) (32,54). In addition, C-Gly (**2c**) undergoes deamination to U-Gly (**2d**) and subsequent dehydation to 5ohC (**7c**) and 5ohU (**7d**). Thus, the pathway leading to glycol products in the case of cytosine includes U-Gly (**2d**), 5ohC (**7c**) and 5ohU (**7d**) (Pathway I).

The quantitative importance of each pathway in the formation of stable products can be compared for thymine, cytosine and 5mC (Table 1). For simplicity, we will only consider the yields for CT-DNA. In the case of thymine, the yield of products arising from the initial addition of •OH to the 5,6-double bond (pathways I and II) leads to a ratio of glycol to hydantoin products of 4:1. In comparison, the ratio was 3.4:1 for cytosine and only 1:1 for 5mC. These results show that the pathway for the formation of glycol products decreases or that the pathway to hydantoin products increases in the case of 5mC. Indeed, the ratio of glycol to hydantoin products from 5mC appears to be even smaller in selected ODN duplexes (Table 1).This changeover in the yield of glycol versus hydantoin products may be attributed to an effect of the C4 substituent (carbonyl or amino group) on the decomposition of intermediate 5(6)-hydroxy-6(5)-hydroperoxides (9,10). For example, a carbonyl group at C4 may favor O–O bond cleavage of the hydroperoxide leading to glycol products (**2**) while an amino group at C4 leads to more efficient C5-C6 bond cleavage of the pyrimidine ring leading to hydantoin products (**3**). The higher yield of hydantoin products from the oxidation of 5mC compared to cytosine may also be due to a change in the percentage of initial •OH addition to C5 and C6, which is 87% to 13% for cytosine, and 65% to 35% for 5mC, respectively (55). An increase in the addition of •OH at C6 may increase the yield of pyrimidine 6-hydroxyl-5-hydroperoxides, which is known, at least for thymidine hydroperoxides, to preferentially decompose into hydantoin products (53).

Additional pathways are proposed to account for the formation of 5hmC (**5a**) and 5fC (**6a**) from the oxidation of 5mC as well as the formation of 5hmU (**5b**) and 5fU (**6b**) from the oxidation of thymine (Scheme 2). The formation of methyl oxidation products likely arises from initial H-atom abstraction from the methyl group by •OH, followed by formation of the corresponding peroxyl radicals (**14**) and hydroperoxides (**15**) in oxygenated aqueous solution. The hydroperoxides of thymine (5-hydroperoxymethyluracil) and that of 5mC (5-hydroperoxymethylcytosine) are fairly stable as 2′-deoxyribonucleosides at room temperature and neutral pH (half-life > 10 h) (53,56). These hydroperoxides (**15**) can decompose by two-electron reduction to give the alcohol (**5**) or by a one-electron process to first give an alkoxyl radical (**16**) that in turn transforms into the alcohol (**5**) by H-atom abstraction (Pathway IVa). Alternatively, the alcohol (**5**) can arise from loss of superoxide radical anion from the peroxyl radical (**14**) giving a carbocation (**17**) that converts to the alcohol by the addition of H_2_O (57) (Pathway IVb). The yield of methyl oxidation products of 5mC and thymine were several fold higher than that estimated by the percentage of H-atom abstraction from the methyl group by •OH for monomers in solution (5–10%). For example, the yield of 5fU and 5hmU was 69% of the total oxidation products of thymine, taking the average of all DNA contexts; in comparison, the yield of 5fC and 5hmC was 57% of the total oxidation products of 5mC. The difference between the 2′-deoxyribonucleosides and duplex DNA contexts is likely due to the greater accessibility of •OH to attack the methyl group of thymine and 5mC, which protrudes into the major groove of double helix DNA. In comparison, the 5,6-double bond of pyrimidine bases is less accessible to •OH because it is buried in the helix interior.

### Sensitivity of damage at C and 5mC

It is well known that the methylation of cytosine in DNA increases the rate of hydrolytic deamination (6–8), the rate of photodimerization with UV light (58), the reactivity of neighboring guanine toward electrophiles (59), and can increase base radical cation hole transfer and damage at this site (60). Although attempts have been made to compare the susceptibility of cytosine and 5mC to •OH-induced oxidation, these attempts fall short because they do not measure the majority of products in biological contexts. In the present study, the susceptibility of C and 5mC toward •OH was determined by the analysis of several oxidation of each base using LC-MS/MS. On the basis of the majority of cytosine and 5mC degradation products at identical sites of ODN duplexes, the methylation of C results in a 1.8-fold increase in the sensitivity of this base to oxidation by •OH. This difference is likely due to the presence of the methyl group that as for thymine is highly susceptible to H-atom abstraction.

### Changes in the distribution of products

The profile of products for each DNA base (5mC, cytosine and thymine) varied substantially within the five ODN duplexes under study (Table 1). These changes can be attributed in part to an effect of the methylation of cytosine on the conformational structure of double-stranded DNA. The incorporation of 5mC in place of cytosine in ODN duplexes under study stabilizes the duplexes as shown by increases in *T*_m_ of 1–3°C of the methylated sequences (Figure 2). The variation of products within different ODN duplexes may also be related to the nature of the base next to damaged site. For instance, there is growing evidence today that a large percentage of •OH-induced DNA damage arises from the reaction of intermediate pyrimidine peroxyl radicals of DNA bases (i.e. structures **9** and **14**, Schemes 1 and 2) with neighboring bases (48,50,52,61–65). Large changes in the yield of guanine oxidation products have recently been observed to depend on the methylation of cytosine and the nature of neighboring bases (66). The proximity of DNA bases to the termini may also play an important role in the formation of products. The results of 5fC and 5fU in ODN duplexes are in good agreement with previous studies using either iron complexes and H_2_O_2_ or radiation-induced •OH as oxidizing agents (67–69).

### Formation of 5mC products in cellular DNA

Several •OH-induced decomposition products of 5mC, cytosine and thymine were measured as a function of radiation dose in cellular DNA (Table 2). Of the 5mC oxidation products, the main radiation-induced modified base was 5hmC followed by 5fC and 5mC-Hyd; in contrast, 5mC-Gly and 5mC-Imid were not detected. From the rates of formation of 5mC products, it is clear that extremely high and lethal doses of ionizing radiation (6 kGy) would be necessary to significantly convert 5mC to 5hmC in cellular DNA; thus an acute dose of ionizing radiation cannot directly induce epigenetic changes at physiologically relevant doses. Interestingly, the rate of formation of 5hmC by ionizing radiation was higher than that of 5fC in cellular DNA, whereas the reverse was true in ODN duplexes or isolated DNA in which 5fC was from 11 to 21-fold higher than 5hmC (Table 1). In addition, the same trend was observed for the corresponding methyl oxidation products of T (5hmU and 5fU). This finding may be related to differences in the concentration of oxygen in cells compared to that in an oxygen saturated solution of DNA. A proportion of intermediate 5-(cytosyl)methyl and 5-(uracilyl) methyl radicals in cellular DNA likely do not react with oxygen because of the low oxygen concentration and thereby they may decay by alternative pathways, for example, by reaction with redox active agents, such as metal ions, which can oxidize the radicals to a carbocation followed by hydration to 5hmC/U products (Pathway IVc). The difference in the ratio of 5hmC/5fC (2.6) and 5hmU/5fU (0.68) may reflect a difference in the rate of reaction of intermediate methyl radicals with oxygen or a difference in the ability of redox agents to oxidize these radicals. Another interesting finding is the amount of 5fU in non-irradiated cells observed in cellular DNA (24.5 lesions per 10^6^ nonmodified DNA bases (Table 2)). This suggests that there may be relatively high levels of endogenous 5fU in the DNA of mouse F98 brain cells. Wang *et al.* (70) previously reported comparable levels of 5fU (20 lesions/10^6^ bases) in most samples of extracted mammalian DNA.

## CONCLUSIONS

We conclude that 5mC is more susceptible by 1.8-fold to •OH-mediated oxidation reactions based on analysis of numerous products in ODN duplexes in which cytosine was replaced with 5mC in identical sequences. The two major oxidation products of 5mC observed in the present study were 5fC and 5mC-Hyd, which together represented from 73 to 89% of the measured products of this base. The addition of •OH to the 5,6-double bond of 5mC yields more hydantoin than glycol products while the comparable reaction with thymine leads to a higher amount of glycol products. The final distribution of stable products ultimately depends on the sequence and structure of ODN duplexes. In addition, there may be different nearest neighbor effects that direct the oxidation of CG and methylated dinucleotides. The nature and importance of tandem base lesions whose formation may be initiated by the generation of pyrimidine hydroperoxyl radicals will require further investiagtion.

## SUPPLEMENTARY DATA

Supplementary Data are available at NAR Online.

SUPPLEMENTARY DATA
