# Supplementary material for: Hydroxyl-radical-induced oxidation of 5-methylcytosine in isolated and cellular DNA
Source: Nucleic Acids Res. 2014 May 22;42(11):7450–60. doi: 10.1093/nar/gku334 (PMC4066766; doi:10.1093/nar/gku334)
Supplement: SUPPLEMENTARY DATA [file supp_42_11_7450__index.html]

Hydroxyl-radical-induced oxidation of 5-methylcytosine in isolated and cellular DNA — Hydroxyl-radical-induced oxidation of 5-methylcytosine in isolated and cellular DNA — SUPPLEMENTARY DATA 

# Hydroxyl-radical-induced oxidation of 5-methylcytosine in isolated and cellular DNA

## SUPPLEMENTARY DATA

**Files in this Data Supplement:**

- SUPPLEMENTARY DATA
