# Supplementary material for: Hydroxyl-radical-induced oxidation of 5-methylcytosine in isolated and cellular DNA
Source: Nucleic Acids Res. 2014 May 22;42(11):7450–60. doi: 10.1093/nar/gku334 (PMC4066766; doi:10.1093/nar/gku334)
Supplement: SUPPLEMENTARY DATA [file supp_gku334_nar-00203-f-2014-File002.doc]

Hydroxyl-radical-induced oxidation of 5-methylcytosine in isolated and cellular DNA

Guru S. Madugundu^1^, Jean Cadet^1,2^ and J. Richard Wagner^1^*

^1^Département de Médecine nucléaire et Radiobiologie, Faculté de Médecine, 3001 12e avenue Nord, Université de Sherbrooke, (Québec) Canada J1H 5N4.

^2^Institut Nanosciences & Cryogénie/DSM, CEA/Grenoble, 38054 Grenoble, France.

* To whom correspondence should be addressed. Tel: 1-819-820-6868 ext. 12717; Email: [richard.wagner@usherbrooke.ca](mailto:richard.wagner@usherbrooke.ca)

Address: J. Richard Wagner Département de Médecine nucléaire et Radiobiologie, Faculté de Médecine, 3001 12e avenue Nord, Université de Sherbrooke, (Québec) Canada J1H 5N4.

**Supporting Information**

Figure S1.1. Melting curve of ODN duplex 1:1 page 3

Figure S1.2. Melting curve of ODN duplex 2:2 page 4

Figure S1.3. Melting curve of ODN duplex 3:4 page 5

Figure S1.4. Melting curve of ODN duplex 3:6 page 6

Figure S1.5. Melting curve of ODN duplex 5:6 page 7

Figure S2.1. MS analysis of 5mC-Gly 2’-deoxyribonucleoside (2a) page 8

Figure S2.2. MRM analysis of 5mC-Gly 2’-deoxyribonucleoside (2a) page 9

Figure S3.1. MS Analysis of Hyd-5mC 2’-deoxyribonucleoside (3a) page 10

Figure S3.2. MRM analysis of Hyd-5mC 2’-deoxyribonucleoside (3a) page 11

Figure S4.1. MS Analysis of Imid-5mC 2’-deoxyribonucleoside (4a) page 12

Figure S4.2. MRM analysis of Imid-5mC 2’-deoxyribonucleoside (4a) page 13

Figure S5.1. MS analysis of 5hmC 2’-deoxyribonucleoside (5a) page 14

Figure S5.2. MRM analysis of 5hmC 2’-deoxyribonucleoside (5a) page 15

Figure S6.1. MS analysis of 5fC 2’-deoxyribonucleoside (6a) page 16

Figure S6.2. MRM analysis of 5fC 2’-deoxyribonucleoside (5a) page 17

**S1.1. Melting curve of ODN duplex 1:1**

5’-CGC GAA TTC GCG-3’

3’-GCG CTT AAG CGC-5’

T_m_ = 64.2 ± 0.3^o^C

Population of single strand is 3.3% and double strand is 96.7% at 23°C.

**S1.2. Melting curve of ODN duplex 2:2**

5’-CGX GAA TTX GCG-3’

3’-GCG XTT AAG XGC-5’; X=5mC

T_m_ = 65.9 ± 0.3^o^C

Population of single strand is 6.7% and double strand is 93.3% at 23°C.

**S1.3. Melting curve of ODN duplex 3:4**

5’-ATA TCG ACG TCG ACG TAT A-3’

3’-TAT AGC TGC AGC TGC ATA T-5’

T_m_ = 66.1 ± 0.4^o^C

Population of single strand is 0.3 and double strand is 99.7% at 23°C.

**S1.4. Melting curve of ODN duplex 3:6**

5’-ATA TCG ACG TCG ACG TAT A-3’

3’-TAT AGX TGX AGX TGX ATA T-5’; X=5mC

T_m_ = 66.8 ± 0.6 ^o^C

Population of single strand is 1.5% and double strand is 98.5% at 23°C.

**S1.5. Melting curve of ODN duplex 5:6**

5’-ATA TXG AXG TXG AXG TAT A-3’

3’-TAT AGX TGX AGX TGX ATA T-5’; X=5mC

T_m_ = 69.9 ± 0.5^o^C

Population of single strand is 0.1 and double strand is 99.9% at 23°C.

**Figure S2.1. MS analysis of 5mC-Gly 2’-deoxyribonucleoside (2a)**

**Figure S2.2. MRM analysis of 5mC-Gly 2’-deoxyribonucleoside (2a)**

**Figure S3.1. MS Analysis of Hyd-5mC 2’-deoxyribonucleoside (3a)**

**Figure S3.2. MRM analysis of Hyd-5mC 2’-deoxyribonucleoside (3a)**

**Figure S4.1. MS Analysis of Imid-5mC 2’-deoxyribonucleoside (4a)**

**Figure S4.2. MRM analysis of Imid-5mC 2’-deoxyribonucleoside (4a)**

**Figure S5.1. MS analysis of 5hmC 2’-deoxyribonucleoside (5a)**

**Figure S5.2. MRM analysis of 5hmC 2’-deoxyribonucleoside (5a)**

**Figure S6.1. MS analysis of 5fC 2’-deoxyribonucleoside (6a)**

**Figure S6.2. MRM analysis of 5fC 2’-deoxyribonucleoside (5a)**
